# Frequent Exertion and Frequent Standing at Work, by Industry and Occupation Group — United States, 2015

**DOI:** 10.15585/mmwr.mm6701a1

**Published:** 2018-01-12

**Authors:** Taylor M. Shockey, Sara E. Luckhaupt, Matthew R. Groenewold, Ming-Lun Lu

**Affiliations:** ^1^Division of Surveillance, Hazard Evaluations, and Field Studies, National Institute for Occupational Safety and Health, CDC; ^2^Division of Applied Research and Technology, National Institute for Occupational Safety and Health, CDC.

Repeated exposure to occupational ergonomic hazards, such as frequent exertion (repetitive bending or twisting) and frequent standing, can lead to injuries, most commonly musculoskeletal disorders (*1*). Work-related musculoskeletal disorders have been estimated to cost the United States approximately $2.6 billion in annual direct and indirect costs (*2*). A recent literature review provided evidence that prolonged standing at work also leads to adverse health outcomes, such as back pain, physical fatigue, and muscle pain (*3*). To determine which industry and occupation groups currently have the highest prevalence rates of frequent exertion at work and frequent standing at work, CDC analyzed data from the 2015 National Health Interview Survey (NHIS) Occupational Health Supplement (OHS) regarding currently employed adults in the United States. By industry, the highest prevalence of both frequent exertion and frequent standing at work was among those in the agriculture, forestry, fishing, and hunting industry group (70.9%); by occupation, the highest prevalence was among those in the construction and extraction occupation group (76.9%). Large differences among industry and occupation groups were found with regard to these ergonomic hazards, suggesting a need for targeted interventions designed to reduce workplace exposure.

NHIS is an annual, in-person, household interview survey of noninstitutionalized, U.S. civilian residents that has been continuously conducted since 1957 with the main purpose of monitoring the health of the U.S. population through assessment of a range of health topics and demographic characteristics.* The NHIS questionnaire contains a set of core questions with Household, Family, Sample Adult, and Sample Child components, which have remained relatively unchanged from 1997 through 2017. In addition, NHIS has sets of questions, known as Supplements, which vary each year depending on new public health data needs. In 2015, CDC’s National Institute for Occupational Safety and Health (NIOSH) sponsored an OHS to collect information on work-related health conditions as well as psychological and physical occupational exposures. The OHS questions were included in the Sample Adult questionnaire, which had a final, unconditional response rate of 55.2%.^†^

To determine industry and occupation, currently employed adult respondents were asked, in reference to the job they were working at during the week before the interview, “What kind of business or industry was this?” and “What kind of work were you doing?” Open-ended responses were recorded as text and subsequently coded by the U.S. Census Bureau into 4-digit codes derived from the 2012 North American Industrial Classification System (NAICS) industry groups and 2010 Standard Occupational Classification (SOC) occupation groups. To improve reliability of the statistical estimates, the detailed 4-digit industry and occupation groups were collapsed into 2-digit industry groups and occupation groups (based on the NAICS and SOC major groups^§^). As part of the OHS, currently employed adults were asked two questions related to the ergonomics of their current job: “How often does your job involve repeated lifting, pushing, pulling, or bending?” and “How often does your job involve standing or walking around?” Responses to these questions were dichotomized into Often/Always and Never/Seldom/Sometimes, to indicate frequent or infrequent exertion or standing, respectively. Responses to these two ergonomics questions were also used to create one dichotomous variable capturing respondents that reported both frequent exertion at work and frequent standing at work.

Among the 36,672 adult NHIS respondents, 19,456 were currently employed and considered for analyses. After excluding 1,615 respondents who worked <20 hours per week, 187 respondents who did not provide adequate information on their hours worked in the previous week, and 190 respondents in military-specific occupations, the final analytic sample included 17,464 respondents (89.8% of the currently employed adult respondents). Sample adults who worked more than 20 hours per week were more likely to be aged <65 years, men, and hold a college degree or higher; however, there was no difference in the distribution of frequent exertion and frequent standing by number of hours worked. Unadjusted prevalence of frequent exertion at work, frequent standing at work, and both frequent exertion and frequent standing at work were calculated by the 20 major industry groups and the 22 major occupation groups. The unadjusted prevalence estimates were obtained using statistical software. All analyses were weighted, and standard errors were adjusted to account for the survey design.

Overall, 39.5% of currently employed adults who work at least 20 hours per week reported both frequent exertion and frequent standing at work (Table 1). The prevalences of frequent exertion at work or frequent standing at work, or both frequent exertion at work and frequent standing at work were highest among men, persons aged 18–29 years, Hispanics, and adults with less than a high school diploma (Table 1).

**TABLE 1:** Weighted prevalence of frequent exertion at work, frequent standing at work, and both frequent exertion and frequent standing at work among adult U.S. workers,* by demographic characteristics — National Health Interview Survey, 2015

Characteristic	Both frequent exertion and frequent standing at work			Frequent exertion at work			Frequent standing at work		
	No. in sample exposed	Weighted no. in population	% (95% CI)	No. in sample exposed	Weighted no. in population	% (95% CI)	No. in sample exposed	Weighted no. in population	% (95% CI)
**Sex**									
Men	3,985	31,887,307	44.1 (42.7–45.5)	4,235	33,946,823	47.0 (45.6–48.3)	6,149	49,783,090	68.8 (67.5–70.2)
Women	2,997	20,897,950	34.0 (32.7–35.4)	3,124	21,864,499	35.6 (34.2–37.0)	5,520	39,328,061	64.0 (62.6–65.4)
**Age group (yrs)**									
18–29	1,682	14,707,666	49.2 (46.7–51.6)	1,738	15,307,794	51.2 (48.7–53.7)	2,593	22,663,742	75.8 (73.7–77.8)
30–44	2,414	17,548,635	39.0 (37.4–40.7)	2,538	18,448,479	41.0 (39.4–42.7)	3,964	29,256,582	65.1 (63.4–66.7)
45–64	2,603	19,130,997	35.7 (34.1–37.4)	2,769	20,480,104	38.3 (36.6–39.9)	4,517	34,073,378	63.7 (62.1–65.2)
≥65	283	1,397,959	26.3 (22.5–30.0)	314	1,574,945	29.6 (25.8–33.4)	595	3,117,450	58.6 (54.4–62.8)
**Race/Ethnicity**									
White, non-Hispanic	4,256	33,402,064	38.4 (37.2–39.7)	4,466	35,094,556	40.4 (39.1–41.7)	7,078	56,412,145	64.9 (63.6–66.1)
Black, non-Hispanic	919	6,736,754	43.4 (40.6–46.2)	971	7,199,190	46.4 (43.6–49.2)	1,555	11,046,554	71.1 (68.6–73.6)
Other race, non-Hispanic	363	2,340,168	25.4 (21.8–29.0)	387	2,562,924	27.8 (24.1–31.4)	691	4,767,859	51.7 (47.7–55.7)
Hispanic	1,444	10,306,271	46.8 (44.4–49.2)	1,535	10,954,652	49.8 (47.4–52.2)	2,345	16,884,593	76.7 (74.7–78.7)
**Education level^†^**									
Less than high school diploma	910	6,425,171	59.5 (56.0–63.0)	967	6,840,231	63.4 (60.1–66.6)	1,269	9,036,138	83.7 (81.4–86.0)
High school diploma/GED	2,138	16,679,503	56.6 (54.5–58.7)	2,249	17,558,718	59.6 (57.4–61.7)	3,000	23,231,939	78.8 (77.1–80.5)
Some college	2,612	19,898,969	47.3 (45.4–49.2)	2,744	21,027,565	50.0 (48.1–51.9)	3,986	30,423,401	72.3 (70.7–73.8)
Bachelor’s degree or higher	1,303	9,513,585	18.7 (17.4–19.9)	1,379	10,116,181	19.8 (18.6–21.1)	3,384	26,079,808	51.2 (49.4–52.9)
**All currently employed adults**	**6,982**	**52,785,257**	**39.5 (38.5–40.5)**	**7,359**	**55,811,322**	**41.7 (40.7–42.7)**	**11,669**	**89,111,151**	**66.6 (65.6–67.6)**

**Abbreviations:** CI = confidence interval; GED = General Educational Development.

* The survey sample consisted of 17,464 U.S. workers aged ≥18 years who worked at least 20 hours per week.

^†^ Education level only shown for persons aged ≥25 years.

Among the 20 major industry groups, the groups with the highest prevalence of both frequent exertion and frequent standing at work were agriculture, forestry, fishing, and hunting (70.9%); construction (67.2%); and accommodation and food services (57.7%) (Table 2). These same three industry groups also had the highest prevalence rates of frequent exertion at work and frequent standing at work considered separately. The finance and insurance industry group had the lowest prevalence rates of all three exposures (Table 2). Among the 22 major occupation groups, the groups with the highest prevalence of both frequent exertion and frequent standing at work were construction and extraction (76.9%); farming, fishing, and forestry (75.5%); and building and grounds cleaning and maintenance (74.0%) (Table 3). These same three occupation groups also had the highest prevalence rates for frequent exertion at work. The food preparation and serving related occupation group (97.2%) had the highest prevalence of frequent standing at work. The computer and mathematical occupation group had the lowest prevalence rate of the combined exposures of frequent exertion and frequent standing at work (4.6%) (Table 3).

**TABLE 2:** Weighted prevalence of frequent exertion at work, frequent standing at work, and both frequent exertion and frequent standing at work among adult U.S. workers,* by industry group — National Health Interview Survey, 2015

Industry group^†^	Both frequent exertion and frequent standing at work			Frequent exertion at work			Frequent standing at work		
	No. in sample exposed	Weighted no. in population	% (95% CI)	No. in sample exposed	Weighted no. in population	% (95% CI)	No. in sample exposed	Weighted no. in population	% (95% CI)
Agriculture, Forestry, Fishing, and Hunting	199	1,168,731	70.9 (63.2–78.5)	213	1,241,068	75.2 (68.0–82.5)	238	1,428,182	86.6 (81.2–92.0)
Construction	741	5,673,721	67.2 (63.5–70.8)	782	5,959,974	70.6 (67.0–74.2)	900	7,041,656	83.4 (80.4–86.3)
Accommodation and Food Services	703	5,272,820	57.7 (53.6–61.7)	712	5,317,174	58.2 (54.1–62.2)	1,093	8,459,753	92.5 (90.6–94.4)
Retail Trade	955	7,504,966	54.6 (51.1–58.2)	977	7,682,555	55.9 (52.4–59.4)	1,403	11,235,663	81.7 (79.3–84.1)
Arts, Entertainment, and Recreation	138	1,165,969	50.1 (41.3–59.0)	143	1,214,309	52.2 (43.8–60.6)	244	1,869,437	80.4 (74.7–86.0)
Health Care and Social Assistance	1,128	8,186,368	45.9 (43.2–48.6)	1,171	8,486,195	47.6 (44.9–50.3)	1,858	13,360,776	74.9 (72.7–77.1)
Administrative and support and Waste management and remediation services	350	2,780,964	45.7 (41.2–50.1)	374	2,955,167	48.5 (43.9–53.1)	539	4,230,331	69.4 (65.6–73.3)
Manufacturing	818	6,742,939	44.7 (41.8–47.7)	872	7,299,479	48.4 (45.4–51.4)	1,198	10,054,756	66.7 (63.7–69.6)
Other service (except Public Administration)	372	2,875,412	44.1 (39.5–48.7)	389	3,003,845	46.0 (41.6–50.5)	624	4,830,228	74.0 (69.8–78.3)
Transportation and warehousing	294	2,238,125	43.7 (38.5–49.0)	352	2,781,765	54.4 (49.3–59.5)	383	2,882,926	56.4 (51.2–61.5)
Wholesale trade	181	1,563,819	40.2 (33.3–47.2)	195	1,683,560	43.3 (36.4–50.2)	274	2,349,503	60.4 (54.3–66.6)
Utilities	54	283,706	27.7 (19.3–36.1)	56	288,971	28.2 (19.8–36.6)	96	593,375	57.9 (47.4–68.5)
Mining	60	217,846	27.0 (19.3–34.6)	63	226,742	28.1 (20.2–36.0)	88	470,569	58.3 (46.0–70.5)
Real Estate and Rental and Leasing	107	745,525	26.2 (20.2–32.2)	110	794,548	27.9 (21.7–34.2)	242	1,773,803	62.4 (55.2–69.6)
Information	84	701,050	23.7 (17.7–29.6)	96	816,293	27.5 (21.2–33.9)	157	1,305,710	44.0 (37.1–51.0)
Public administration	218	1,594,215	23.0 (19.6–26.4)	234	1,706,227	24.6 (21.1–28.1)	525	3,794,816	54.8 (50.4–59.2)
Education services	390	2,698,347	22.7 (19.9–25.5)	402	2,778,152	23.4 (20.5–26.2)	1,187	8,599,529	72.3 (69.4–75.2)
Professional, scientific, and technical services	134	1,026,452	9.8 (7.6–12.0)	153	1,159,929	11.1 (8.8–13.3)	404	3,149,955	30.1 (26.5–33.6)
Finance and Insurance	55	342,473	5.0 (3.2–6.8)	64	413,560	6.0 (3.9–8.2)	209	1,651,592	24.1 (20.3–27.9)
**All currently employed adults**	**6,982**	**52,785,257**	**39.5 (38.5–40.5)**	**7,359**	**55,811,322**	**41.7 (40.7–42.7)**	**11,669**	**89,111,151**	**66.6 (65.6–67.6)**

**Abbreviation:** CI = confidence interval.

* The survey sample consisted of 17,464 U.S. workers aged ≥18 years who worked at least 20 hours per week.

^†^ The Management of Companies and Enterprises industry group was removed from the results because the cell size was <10 and did not meet the National Center for Health Statistics’ standards of reliability.

**TABLE 3:** Weighted prevalence of frequent exertion at work, frequent standing at work, and both frequent exertion and frequent standing at work among adult U.S. workers,* by occupation group — National Health Interview Survey, 2015

Occupation group	Both frequent exertion and frequent standing at work			Frequent exertion at work			Frequent standing at work		
	No. in sample exposed	Weighted no. in population	% (95% CI)	No. in sample exposed	Weighted no. in population	% (95% CI)	No. in sample exposed	Weighted no. in population	% (95% CI)
Construction and Extraction	685	4,856,232	76.9 (73.2–80.6)	718	5,077,403	80.4 (76.8–84.1)	793	5,739,639	90.9 (88.7–93.2)
Farming, Fishing, and Forestry	129	731,178	75.5 (65.3–85.8)	133	749,387	77.4 (67.3–87.4)	147	888,366	91.7 (86.9–96.6)
Building and Grounds Cleaning and Maintenance	518	3,495,764	74.0 (69.9–78.1)	532	3,605,331	76.3 (72.3–80.3)	658	4,332,921	91.7 (88.7–94.7)
Installation, Maintenance, and Repair	436	1,290,688	73.0 (68.4–77.6)	451	3,611,498	75.5 (71.0–79.9)	516	4,238,754	88.6 (85.1–92.1)
Food Preparation and Serving Related	587	4,277,608	65.7 (61.1–70.2)	591	4,304,915	66.1 (61.5–70.7)	853	6,336,456	97.2 (96.1–98.4)
Production	704	5,615,533	65.2 (61.5–69.0)	738	5,884,574	68.3 (64.7–72.0)	940	7,310,817	84.9 (82.2–87.6)
Healthcare Support	285	1,965,904	62.2 (56.2–68.1)	291	2,010,000	63.6 (57.6–69.5)	395	2,769,227	87.6 (83.5–91.7)
Transportation and Material Moving	540	4,244,701	55.2 (51.0–59.5)	642	5,187,351	67.5 (63.4–71.6)	648	5,182,206	67.4 (63.4–71.4)
Healthcare Practitioners and Technical	527	4,272,338	53.1 (49.2–57.1)	543	4,375,067	54.4 (50.3–58.5)	874	6,955,460	86.5 (84.1–88.9)
Personal Care and Service	278	2,072,693	52.2 (46.1–58.3)	290	2,135,012	53.8 (47.7–59.8)	467	3,448,249	86.8 (83.3–90.4)
Sales and Related	657	5,313,669	39.3 (35.7–42.9)	675	5,484,798	40.6 (37.0–44.2)	1,194	10,006,503	73.9 (70.9–76.8)
Protective Service	119	907,995	35.0 (28.2–41.8)	123	925,096	35.6 (28.7–42.6)	280	2,198,270	84.7 (79.5–89.8)
Education, Training, and Library	267	2,057,303	25.2 (21.5–28.9)	274	2,104,193	25.8 (22.1–29.5)	891	6,717,390	82.2 (79.2–85.3)
Office and Administrative Support	528	3,847,255	24.3 (21.8–26.8)	567	4,166,405	26.3 (23.7–28.9)	996	7,528,768	47.5 (44.8–50.2)
Arts, Design, Entertainment, Sports and Media	74	579,465	22.8 (16.9–28.7)	80	631,237	24.8 (18.9–30.8)	171	1,145,550	45.1 (38.3–51.8)
Management	418	3,258,402	22.5 (19.9–25.1)	444	3,441,037	23.7 (21.1–26.4)	965	7,543,484	52.0 (49.0–55.0)
Community and Social Services	52	423,039	15.7 (10.0–21.5)	53	444,643	16.5 (10.7–22.4)	217	1,448,680	53.9 (47.1–60.7)
Life, Physical, and Social Science	31	176,272	12.1 (6.1–18.0)	34	220,958	15.1 (8.3–21.9)	107	734,098	50.2 (40.9–59.5)
Architecture and Engineering	35	337,561	10.2 (6.1–14.3)	39	366,910	11.1 (6.9–15.3)	144	1,318,074	39.8 (33.4–46.3)
Business and Financial Operations	76	594,080	7.9 (5.7–10.1)	89	696,504	9.2 (6.9–11.6)	242	1,967,945	26.1 (22.5–29.7)
Computer and Mathematical	29	232,386	4.6 (2.6–6.6)	41	336,596	6.7 (4.1–9.2)	116	949,751	18.8 (14.8–22.8)
Legal	7	32,566	—^§^	11	52,407	3.1 (0.8–5.4)^†^	55	350,543	20.6 (13.5–27.6)
**All currently employed adults**	**6,982**	**52,785,257**	**39.5 (38.5–40.5)**	**7,359**	**55,811,322**	**41.7 (40.7–42.7)**	**11,669**	**89,111,151**	**66.6 (65.6–67.6)**

**Abbreviation:** CI = confidence interval.

* The survey sample consisted of 17,464 U.S. workers aged ≥18 years who worked at least 20 hours per week.

^†^ Estimate has a relative standard error >30% and <50% and should be used with caution because it does not meet the National Center for Health Statistics’ standards of reliability.

^§^ Estimate had a cell size <10 and was removed from the results because it did not meet the National Center for Health Statistics’ standards of reliability.

## Discussion

This is the first CDC report to evaluate exposure to frequent exertion and frequent standing at work among U.S. employed adults in all industries and occupations. The prevalence of exposure to both of these ergonomic hazards was higher among agricultural and construction workers than among workers in all other industries. A previous study using the U.S. Department of Labor’s Occupational Information Network database found that of 10 detailed occupation categories evaluated with regard to self-reported bending or twisting at work, half were construction-related, which is consistent with the findings from this study (*4*). In addition, previous research using NHIS data that evaluated musculoskeletal disorders among agricultural workers found that low back pain was the most prevalent musculoskeletal disorder. That study also found that agricultural workers had a significantly higher prevalence of upper extremity pain compared with all other industries (*5*). Research has shown that agricultural and construction work are physically demanding, as these industries often require manual material handling, repetitive exertions, awkward body postures, and use of machinery that causes whole body vibration (*4*–*7*).

Approximately two thirds of all workers reported frequent standing at work. The industry and occupation groups that reported high prevalence rates of frequent exertion (e.g., farming, construction, and food services) also tended to report high prevalence rates of frequent standing, possibly because bending, pushing, pulling, and lifting commonly co-occur with standing. Several industry and occupation groups, such as education and protective services, reported a high prevalence of frequent standing at work with a low prevalence of frequent exertion at work compared with other industry and occupation groups.

Recent studies have emphasized health risks associated with excessive sitting during the workday (*8*); however, excessive standing on the job also has been linked to adverse health outcomes (*9*). A systematic review of peer-reviewed articles on musculoskeletal symptoms and occupational standing as the main exposure variable found that occupational standing is associated with low back pain; however, associations with lower and upper extremity symptoms were inconclusive (*9*). More research is needed to understand how to balance time spent sitting and standing while at work.

The findings in this report are subject to at least four limitations. First, because NHIS data are cross-sectional, it is not possible to make causal inferences. Second, because NHIS data are self-reported, they are subject to recall or social desirability bias. Third, the intermediate exposure categories (Often, Sometimes, and Seldom) rely on subjective assessment of frequency. Finally, collapsing the detailed industry and occupation groups into the major industry and occupation groups might have aggregated employees with different working conditions.

*Healthy People 2020* has an objective to “reduce rate of injury and illness cases involving days away from work due to overexertion and repetitive motion,” by at least 10%.^¶^ NIOSH has developed educational resources on a variety of ergonomic issues.** For example, NIOSH provides a demonstration guide on ergonomic principles including how to maintain neutral postures when working, how to select the appropriate hand tools, and how to prevent fatigue failure of the vertebrae. In addition, NIOSH offers ergonomic guidelines for manual material handling, a primer for creating a workplace ergonomic programs, and ergonomic interventions by specific industry, including agriculture and construction.^††^ Because ergonomic hazards are risk factors for work-related musculoskeletal disorders, continued research is necessary to develop a better understanding of these hazards and to create interventions aimed at reducing them (*2*,*8*–*10*).

**Summary**

**What is already known about this topic?**

Occupational ergonomic hazards are risk factors for negative health outcomes such as musculoskeletal disorders. Previous research has found that employees in the agricultural and construction sectors experience high rates of musculoskeletal disorders and other injuries because of the physical nature of the work and has also found that workers in the construction and agricultural sectors have high prevalence rates of exertion including bending, lifting, pushing, and pulling.

**What is added by this report?**

Analysis of data from the National Health Interview Survey to examine two ergonomic hazards among currently employed adults who work at least 20 hours per week in 20 major industry groups and 22 major occupation groups found a 41.7% prevalence of frequent exertion (repeated lifting, pushing, pulling, or bending) at work and a 66.6% prevalence of frequent standing at work. A wide range in prevalence for these ergonomic hazards was observed among the industry and occupation groups.

**What are the implications for public health practice?**

Large differences in prevalence of frequent exertion at work and frequent standing at work exist among the major industry and occupation groups. Identification of workers with the highest prevalences of exposure to these two ergonomic hazards can inform the targeting of interventions.

## Footnotes

*
https://www.cdc.gov/nchs/nhis/about_nhis.htm .

† ftp://ftp.cdc.gov/pub/Health_Statistics/NCHS/Dataset_Documentation/NHIS/2015/srvydesc.pdf.

§ https://www.census.gov/cgi-bin/sssd/naics/naicsrch?chart=2012 and https://www.bls.gov/soc/major_groups.htm.

¶
https://www.healthypeople.gov/2020/topics-objectives/topic/occupational-safety-and-health/objectives .

†† https://www.cdc.gov/niosh/docs/2001-111/ and https://www.cdc.gov/niosh/docs/2007-122/.
